# Research Progress on *PCR* (Plant Cadmium Resistance) Genes in Plants

**DOI:** 10.3390/biology14091163

**Published:** 2025-09-01

**Authors:** Hongzheng Li, Shuyu Liu, Zhiqi Chen, Linyan Qiu, Xianfeng Wang, Xianhui Kang, Jujuan Gao, Pingping Guo, Wenbo Lin, Chenglang Pan

**Affiliations:** 1 College of Resources and Environment, Fujian Agriculture and Forestry University, Fuzhou 350002, China; 15737754821@163.com (H.L.); 15086303964@163.com (S.L.); 15185960844@163.com (L.Q.); 2 Fujian Key Laboratory on Conservation and Sustainable Utilization of Marine Biodiversity, Fuzhou Institute of Oceanography, College of Geography and Oceanography, Minjiang University, Fuzhou 350108, China; czq0906shiyu@163.com (Z.C.); w1294156176@163.com (X.W.);; 3 College of Life Sciences, Fujian Agriculture and Forestry University, Fuzhou 350002, China; 4 School of Future Technology, Fujian Agriculture and Forestry University, Fuzhou 350002, China; 5 Fujian Minjiang River Estuary Wetland National Nature Reserve Administrative Office, Fuzhou 350200, China; gaojujuan996@163.com (J.G.);

**Keywords:** environmental pollution, cadmium resistance genes, cadmium resistance mechanism of plants, functional differentiation, PLAC8 motif

## Abstract

Heavy metal pollution is becoming increasingly severe, and cadmium (Cd) is one of the most threatening pollutants. The PCR (Plant cadmium resistance) gene encodes a class of small transmembrane proteins containing the PLAC8 motif, which confer cadmium tolerance to plants through multiple mechanisms such as efflux, compartmentalization, chelation, and antioxidant activity, and regulate fruit size and ion homeostasis. This study systematically integrated the *PLAC8*/*PCR* gene families from mosses, monocots, and dicots, revealing their structural and functional relationships, evolutionary trajectories, and functional diversification patterns through phylogenetic and motif analyses, providing a theoretical basis for cadmium-resistant breeding and environmental remediation. Future research should further integrate multi-omics and gene editing technologies to deeply elucidate the transport mechanism of the PCR protein pentamer and the functional differences of key motifs (CCXXXXCPC, CCXXCAL, and CCXXG), and conduct field trials to assess their ecological safety and crop application potential.

## Simple Summary

*PCR* (Plant cadmium resistance) genes are a kind of small transmembrane protein containing a PLAC8 motif, which is characterized by two transmembrane α-helices. It is related to two distinct functions: one is to control fruit size and weight, and the other is to endow plants with cadmium resistance and ion homeostasis, which plays an important role in heavy metal transport. In this paper, the PLAC8 motif-related genes of moss, monocotyledons, and dicotyledons were clustered, and the specific sites were found. The structure, function, evolution, and functional differentiation were analyzed, which provides a theoretical basis for cadmium-resistant plant breeding, environmental governance, and sustainable agriculture, and could help solve the problem of heavy metal pollution.

## 1. Introduction

In the natural world, plants confront a multitude of environmental stressors, with heavy metal pollution emerging as one of the most formidable threats [1]. Cadmium pollution, in particular, stands out as a quintessential example, exerting profound negative effects on ecosystems and agricultural productivity [2]. Cadmium, as a non-essential nutrient element, possesses robust migratory abilities [3]. It is readily absorbed and accumulates within plant tissues, disrupting the normal uptake and translocation of mineral nutrients by plants and markedly inhibiting their growth and development. Even more concerningly, the accumulation of cadmium in plants cannot be metabolized away and instead is transmitted and concentrated through the food chain, ultimately posing severe and irreversible health risks to humans, who occupy the apex of the food chain [2]. Using rice as an example, as one of the world’s crucial food crops, it has been under persistent threat of cadmium pollution, which not only impacts rice yield and quality but also has direct implications for food security and human health [4].

In response to cadmium stress, plants have developed an array of intricate physiological and molecular mechanisms [5]. These mechanisms primarily encompass strategies such as the regulation of photosynthesis, the efflux of cadmium ions, and compartmentalization [6]. Specifically, some plants actively secrete cadmium ions outside the cell through efflux mechanisms, thereby reducing cadmium accumulation within the body [7]; meanwhile, others utilize compartmentalization mechanisms to sequester cadmium ions in specific organelles such as vacuoles, reducing their toxic effects on biological macromolecules in the cytoplasm [8]. For example, barley (Hordeum vulgare), a common food crop, exhibits strong cadmium tolerance [9]. Research has demonstrated that under cadmium stress, barley cells actively transport cadmium ions into the vacuole via transporters located on the vacuolar membrane, achieving compartmentalization. The cadmium concentration accumulated within the vacuole can reach up to 38 times that of the extracellular concentration [10]. Under cadmium stress, the distribution of Cd in the root cell wall of wheat increased by 54.7%, and the content of lignin, pectin, and hemicellulose also increased. This indicates that cell wall compartmentalization plays an important role in the cadmium detoxification mechanism [11].

**Figure 1:**
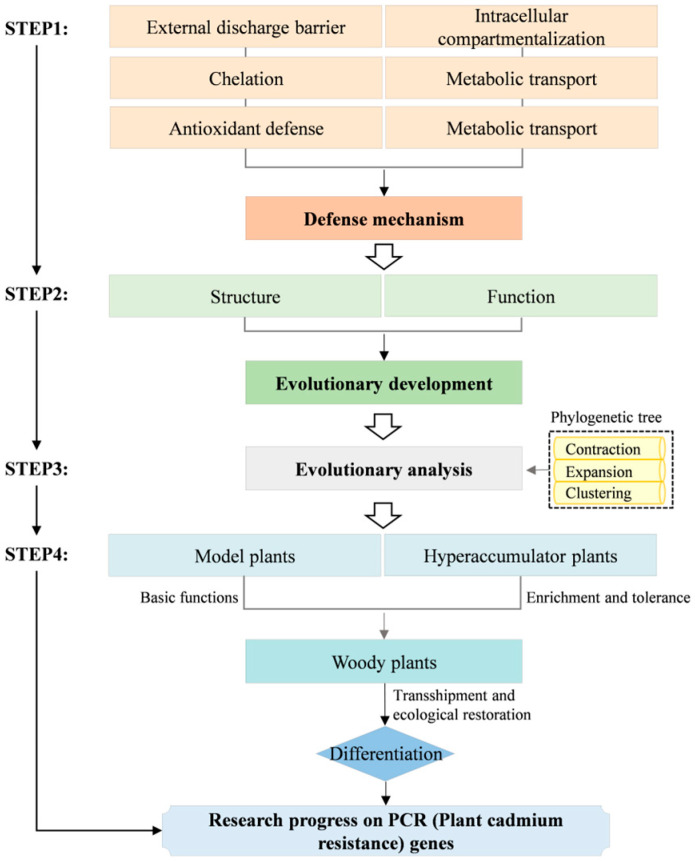
Overview of the PCR (Plant cadmium resistance) gene roadmap.

In recent years, researchers have identified numerous cadmium-resistant genes that, through unique signaling pathways or transduction mechanisms, target specific genes or functional elements and mitigate damage induced by reactive oxygen species (ROS) through their interactions [12,13]. *PCR* (Plant cadmium resistance) genes represent a class of small transmembrane proteins that harbor a PLAC8 motif with an uncharacterized function, playing a pivotal role in cadmium resistance processes [14]. The PLAC8 (Placental-specific 8) domain was originally discovered in 2003 by Galaviz-Hernandez et al. in mammalian placental spongiotrophoblast proteins [15]. In the plant kingdom, the first PLAC8 protein in Arabidopsis thaliana was named PCR (PF04749), belonging to a class of small membrane proteins [16]. In 2004, Song et al. screened the cadmium-sensitive yeast strain Δycf1 and discovered a new protein that confers cadmium tolerance to these yeast cells, naming the corresponding gene AtPCR1. Through comparative analysis of the Arabidopsis thaliana genome, a total of 11 proteins homologous to AtPCR1 were identified. Notably, AtPCR2 differs from AtPCR1 in the length of its N-terminal domain sequence. Based on this observation, these proteins can be categorized into three distinct branches. The first branch contains only AtPCR10; the second branch includes seven members, none of which have been characterized yet; and the third branch includes gene AtPCR 1,2,3,11 [16]. Subsequently, numerous *PCR* genes have been discovered in various other plants such as maize [17], rice [18], and rapeseed [19]. Studies have shown that *PCR* genes possess unique cadmium-resistant substrates and effectively resist cadmium toxicity through mechanisms such as efflux, chelation, transport, and compartmentalization [20,21,22,23,24,25], significantly enhancing plant cadmium tolerance. In light of the importance and potential applications of *PCR* genes in plant cadmium resistance mechanisms, coupled with our recent novel findings on the functional and structural analysis of the *PCR* gene family, this article endeavors to provide a comprehensive review of research advancements regarding the role of *PCR* genes in plant cadmium resistance (Figure 1). By thoroughly exploring their structure, function, evolutionary trajectory, and functional differentiation across various plant types, this article endeavors to offer a fresh perspective on phytoremediation of heavy metal contamination in plants. Additionally, it aims to provide theoretical underpinnings for ecological restoration, the breeding of cadmium-resistant crop varieties, and related molecular application technologies, thereby fostering further advancements in this research domain.

## 2. The Role of *PCR* Genes in Plant Defense Mechanisms Against Cadmium Stress

**Figure 2:**
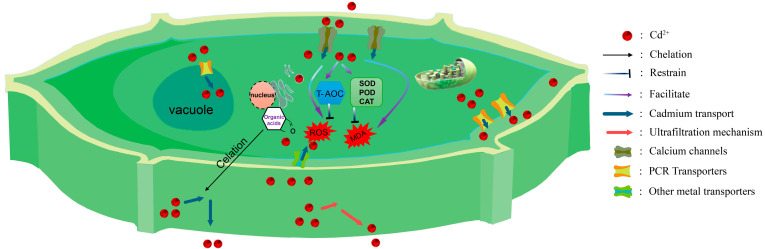
Defense mechanism of *PCR* genes in response to cadmium stress. Reduction of cadmium transport into cells through the ultrafiltration mechanism of plant cell walls; extracellular excretion; chelation of cadmium with plant organic acids; compartmentalization of vacuoles, transporting cadmium into vacuoles via PCR transport proteins on the vacuole membrane; enhancing the antioxidant system, such as superoxide dismutase (SOD), peroxidase (POD), and catalase (CAT), to mitigate the damage caused by ROS and MDA to plant cells.

In response to cadmium stress, plants have evolved a complex defense system to mitigate the harm caused by cadmium. This system spans multiple levels, from the extracellular environment to the intracellular space [12]. Specifically, it manifests as various strategies, including secretion of organic acids by the roots to reduce the availability of cadmium ions, compartmentalization mechanisms of the organelles, and metabolic transformations within the plant [26,27,28]. The synergistic operation of these defense mechanisms enables plants to alleviate, to some extent, the growth pressure imposed by cadmium stress. *PCR* genes play a crucial role in these defense mechanisms, and each will be introduced individually in the following sections (Figure 2).

### 2.1. Extracellular Exclusion Barrier

The extracellular efflux mechanisms by which plants respond to cadmium stress primarily include cell wall adsorption, active efflux via membrane transport proteins, and the formation of apoplast barriers [29]. The cell wall, with its rich structural components such as cellulose, hemicellulose, and pectin, can adsorb a large amount of metal ions, thereby significantly reducing the activity of cadmium ions in the surrounding environment and alleviating their toxic effects on cells [30]. Meanwhile, various transport proteins on the cell membrane, such as H^+^-ATPase, can actively transport cadmium ions from the cell interior to the extracellular space, achieving effective cadmium ion efflux from the cell to maintain a low cadmium environment within the cell [31]. BnPCR10.1 is highly expressed in multiple organs, and its expression in yeast cells enhances the efflux of Cu and Cd. Transgenic Arabidopsis experiments have shown that overexpression of BnPCR10.1 reduces Cu concentration in roots and Cd concentration in both aboveground parts and roots, while decreasing root uptake of Cu^2+^ and Cd^2+^, indicating that BnPCR10.1 primarily detoxifies Cu and Cd through an efflux mechanism [19]. Building on this, plants further limit the diffusion efficiency of cadmium ions into vascular tissues by regulating the formation of the apoplast barrier, thereby reducing cadmium transport and accumulation within the plant, providing additional protection. The *PCR* gene plays a key role in this process by regulating the formation of the apoplast barrier, limiting the diffusion efficiency of cadmium ions into vascular tissues, and consequently reducing cadmium transport and accumulation within the plant [32].

### 2.2. Intracellular Compartmentalization

Vacuoles play a central role in the process of intracellular compartmentalization. The transporters on the vacuolar membrane can actively transport cadmium ions entering the cytoplasmic matrix to the interior of the vacuole, realizing the compartmentalization of cadmium ions, thus protecting biological macromolecules in the cytoplasmic matrix [33]. This process not only reduces the toxic effects of cadmium ions on biological macromolecules in the cytosol but also maintains ionic homeostasis within the cell [21]. Oligomerization is the process by which proteins assemble into stable polymeric structures through the specific assembly of multiple subunits. This structure is crucial for maintaining the conformation stability of proteins and performing specific biological functions. Based on the number of subunits, oligomers can be further classified into dimers, trimers, tetramers, and higher-order polymer forms [34]. In the model plant Arabidopsis thaliana, the *AtPCR1* gene encodes a small transmembrane protein. Structural topology predictions and experimental evidence consistently indicate that this protein exists in a pentameric form, and this pentameric conformation is essential for its physiological function. As a potential cation transporter or channel, the pentameric structure of AtPCR1 may provide the structural basis for efficient transmembrane transport of Cd^2+^ through synergistically formed transmembrane pores and specific metal ion binding sites, thereby achieving vacuolar sequestration of cadmium ions, reducing its free concentration in the cytoplasmic matrix, alleviating the toxic effects of cadmium on cellular components, and enhancing plant tolerance to cadmium stress [17]. Similarly, many ion channels or transporters in plants also rely on specific oligomeric states to achieve functional optimization [35]. For example, members of the Arabidopsis cation/H^+^ antiporter (CAX) family (CAX1 and CAX3) typically exist as dimers or tetramers [36]. This oligomeric conformation helps stabilize the precise arrangement of transmembrane domains, optimize the transport efficiency of substrate ions (such as Ca^2+^ and Mg^2+^), and maintain intracellular ion homeostasis. The pentameric structure of AtPCR1 exhibits high functional consistency with the oligomeric characteristics of CAX family proteins, both of which enhance ion transport activity through higher-order structures, providing structural adaptability for plants under heavy metal stress.

### 2.3. Chelation

In the face of cadmium stress, plant roots reduce the availability of cadmium ions by secreting a large number of organic acids (such as citric acid, malic acid, and oxalic acid) [37,38,39]. These organic acids can form stable chelates with cadmium ions in soil, thus significantly reducing the concentration of free cadmium ions in solution [30]. In addition, plants also utilize chelation to immobilize and sequester cadmium ions within cells, which is a key component of plant cadmium tolerance mechanisms [40]. This chelation process not only alleviates the direct toxicity of cadmium ions to intracellular biomacromolecules but also provides prerequisites for subsequent detoxification mechanisms such as transport and compartmentalization [41]. PCR transmembrane proteins play an auxiliary role in the formation of metal transport channels. As an auxiliary transporter, its unique structure of two transmembrane helices (TM helices) may play a key “bayonet” role. According to the hydrophilic and hydrophobic distribution of amino acid residues, these regions may selectively adsorb and guide heavy metal ions through the channel, thus effectively assisting the transport process of metal ions [42].

### 2.4. Metabolic Transport

The expression pattern of *PCR* genes in plants exhibits tissue specificity, which influences their differential effects on the growth of different plant parts. In Arabidopsis, the *AtPCR1* gene is expressed in stems, leaves, and flowers but not in roots; conversely, the *AtPCR2* gene shows the highest expression in roots, with relatively weaker expression in leaves, stems, and flowers. This specific expression pattern directly influences the metabolic transport of metal ions: knocking out the *AtPCR2* gene leads to growth abnormalities in plants, while its overexpression enhances plants’ tolerance to cadmium and significantly affects their growth, indicating that AtPCR2 plays a key role in cadmium metabolism and transport in plants [42,43]. The Casparian strip, a ribbon-like structure on the endodermal cells of roots, plays a crucial role in preventing the diffusion of cadmium ions from the apoplast to vascular tissues [44]. The *OsPCR1* gene in rice shows a cadmium resistance function consistent with the Kjeldahl band, and its expression in the root system can limit the loading of cadmium ions into the xylem, thus reducing the transport of cadmium to the shoot [32]. The overexpression of wheat *PCR* genes in plant lines increased Cd tolerance and enhanced the ability to translocate Cd from roots to shoots. By isolating Cd in stem and leaf tissues, direct toxicity to the root system was reduced, demonstrating its involvement in the metabolic transport of heavy metal ions and maintaining the structure and function of the root system, which has positive implications for wheat growth [45].

### 2.5. Antioxidant Defense

Cadmium can be absorbed by plants after entering the soil, which will lead to an increase in reactive oxygen species (ROS) levels in plant cells. ROS mainly include superoxide anion (O_2_^−^·), hydrogen peroxide (H_2_O_2_) and hydroxyl radical (·OH) [3,46,47]. Under normal conditions, ROS within plant cells are efficiently scavenged by the antioxidant system [48]. However, under cadmium stress, the production rate of ROS exceeds the scavenging rate, leading to their accumulation within cells and the induction of oxidative stress [49]. As transmembrane transport proteins, *OsPCR1* and *OsPCR3* help plants maintain redox balance by affecting the activity or expression of antioxidant enzymes to alleviate ROS damage to cells [50]. These changes in antioxidant enzyme activities indicate that the antioxidant defense system of plants will make corresponding adjustments and responses to maintain the redox balance in cells under different degrees of cadmium stress.

### 2.6. Transcriptional Regulation Mechanism

The transcriptional regulation mechanism is of central significance in plants’ responses to heavy metal stress. It integrates upstream signal sensing and downstream functional protein activation through fine regulation of *PCR* gene expression to achieve efficient detoxification and tolerance of plants to heavy metal ions. It is a key bridge connecting environmental signals and gene response [51,52]. There are many heavy metal responsive elements in the promoter region of the *AtPCR* gene, such as MRE [53], which can be activated synergistically with the transcription factors bZIP and MYB to ensure their rapid up-regulation of expression under cadmium stress [54]. In addition, *PCR* action elements can be divided into three hormone response, environmental stress, and growth and development-related categories, among which the response elements responding to environmental stress are the most important (Supplementary Table S1). When plants face cadmium and other heavy metal stress, the *AtPCR* promoter can target many heavy metal response transcription factors, which bind to the promoter region, and then regulate the expression of *AtPCR* genes [55]. Under cadmium stress, targeting transcription factors through promoters can achieve precise regulation of *AtPCR* gene expression, thus affecting upstream and downstream signaling pathways related to cadmium tolerance in plants, and enhancing the adaptability of plants to cadmium stress. For example, *PyWRKY71* plays a role in heavy metal stress. The chlorophyll content of transgenic poplar leaves is higher than that of WT, and the activities of POD, SOD, CAT, and MDA) are increased, enhancing the tolerance [56]. The *PCR* promoter contains this binding site, which may regulate upstream and downstream pathways and activate the expression of GSH and PCs pathway-related genes. MYB4, a member of the R2R3 subfamily of MYB-domain proteins, regulates cadmium tolerance by enhancing protection against oxidative damage and increases the expression of *PCS1* and *MT1C* in Arabidopsis thaliana This loss of function mutant line (atmyb4) is hypersensitive to Cd stress [57]. *bHLH104* loss of function mutants are sensitive to Cd stress, and Cd tolerance is enhanced after overexpression of *bHLH104*. In other words, *bHLH104* positively regulates Cd tolerance and Fe-deficient tolerance in Arabidopsis [58]. They were all targeted by *PCR* genes, and there was a cadmium-responsive signaling pathway.

## 3. The Structure, Function, and Evolutionary Progression of PCR

**Figure 3:**
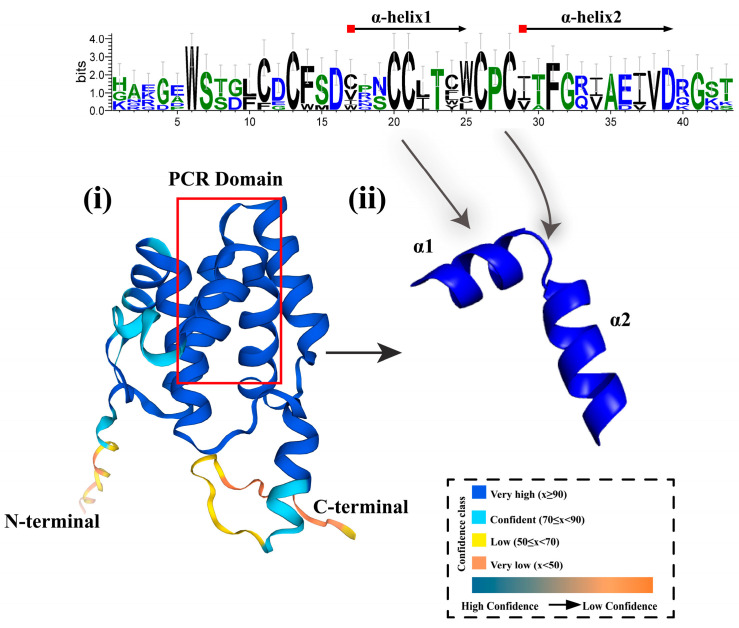
Analysis of Plant *PCR* gene Structure. The red square indicates the location of the CCXXXXCPC motif, and the Web diagram is calculated from Arabidopsis thaliana PCRs. (i) represents the substrate recognition region of the PCR protein; (ii) indicates the protein position corresponding to the two helices. The model was created using AlphaFold3, with different colors indicating varying degrees of conservation.

The *PCR* gene family encodes proteins containing the PLAC8 motif, showing significant amino acid length diversity in different organisms, ranging from 108 to 557 amino acids, and is dominated by short sequence members, most of which are less than 200 amino acids [59,60]. Specifically, in animals, the amino acid length of these proteins is mostly in the range of 108 to 144, while homologous members in fungi usually contain 148 to 157 amino acids [61]. In plants, PLAC8 possesses two conserved domains that are specific to animal proteins [42]. Arabidopsis thaliana (Arabidopsis thaliana) PCR family members such as AtPCR2 encode proteins containing two exons, with an open reading frame length of 663 bp, encoding 220 amino acid residues, a molecular weight of approximately 24.72 kDa, and an isoelectric point of 5.81. Sequence analysis revealed that the amino acid regions from positions 28 to 48 and 51 to 71 of AtPCR2 form two transmembrane domains [16]. Compared with other PCR family members, AtPCR2 exhibits unique characteristics in terms of expression patterns and subcellular localization [32]. AtPCR2 is expressed in both roots and aboveground parts, while AtPCR1 is only expressed in aboveground parts. This difference may influence its functional role in zinc transport and detoxification processes. Additionally, the amino acid sequences near the transmembrane domains of AtPCR2 differ from those of other PCR family members, which may affect its interactions with other proteins as well as its affinity for zinc ions and transport efficiency. Furthermore, members 1, 2, 3, 9, 10, and 11 of the AtPCR family contain the CCXXXXCPC motif, which is important for substrate recognition. This motif includes a linker region with two helices and plays a key role in heavy metal or divalent cation transport, whereas AtPCR7 carries the ACXXXXCPC motif, and other members possess the XPC motif [17]. When the CC in the CCXXXXCPC motif of AtPCR2 undergoes a CL mutation, it affects substrate recognition. In addition to metal translocation, PCR also plays a role in Ca^2+^ transport [62]. BjPCR1 is a Ca^2+^ efflux transporter that efficiently promotes radial transfer of Ca^2+^ in roots and facilitates Ca^2+^ translocation to shoots [63]. Specifically, the Ca^2+^ efflux activity of the Bjpcr1 knockdown cell line was lower than that of wild type (WT), while the Ca^2+^ transport activity of yeast membrane vesicles expressing bjpcr1 increased, and bjpcr1 exported Ca^2+^ to exchange with three protons. The overall data support the hypothesis that bjpcr1 is an output factor required for Ca^2+^ translocation from root epidermis to internal cells and buds. More importantly, the C-terminal region of this protein contains the PLAC8 motif, which includes a transmembrane region, forming the structural basis for its function as an ion channel. Members of the PCR family possess two transmembrane α-helices (Figure 3), indicating that they are membrane-associated or membrane-intrinsic proteins. To further investigate the structural and functional mechanisms, as well as how the CCXXXXCPC motif interacts with heavy metal ions, Guo et al. used the known Mg^2+^ transporter CorA as a reference and concluded that CorA mediates Mg^2+^ uptake and forms homopentamers, and its structure has been confirmed by crystallization. Based on the characteristics of CorA, a structural model of AtPCR1 was constructed, showing that AtPCR1 has the potential to form pentamers and may function as a cation transporter or channel [17]. Furthermore, studies have found that CL mutations in the CCXXXXCPC motif may affect substrate recognition [42]. This may be due to changes in the hydrophilicity and hydrophobicity of amino acids, leading to changes in the pentamer and resulting in functional differences to a certain extent. Although existing research provides evidence for PCR proteins as functional transporters, further verification is still needed, such as through protein–liposome transport experiments or in-depth structural studies to confirm their transport function and rule out the possibility of them merely assisting in the transfer of divalent cations as chaperone molecules.

**Figure 4:**
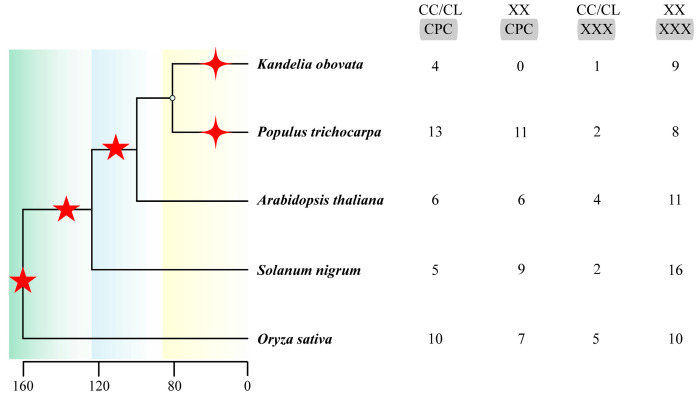
Differentiation times of different species and the number of *PCR* gene substrates. The tree diagram on the left depicts specific fossil time points, highlighting a significant divergence and amplification over the past two hundred thousand years. The pentagrams indicate whole-genome events, while the stars signify tandem duplications. On the right side, we have the substrate counts for the five species, derived from statistical literature and computations.

Genomic duplication events, such as whole-genome duplication (WGD) and whole-genome triplication (WGT), drive the continuous evolution of gene families [64,65]. The complete plant genome sequence allows researchers to analyze the function of PCR. The two conserved domains of the PCR motif, with only the CCXXXXCPC motif undergoing substrate variation, significantly affect plant cadmium resistance. The second domain, QXXRELK, was validated by point-mutation experiments and found to have no significant impact on cadmium resistance [16]. Although QXXRELK may be involved in protein–protein interactions or subcellular localization rather than direct cadmium binding, in the PLAC8 protein, metal ion coordination functions are primarily mediated by the CCXXXXCPC motif [7]. Here, we summarize the PCR members in model plants, enriched plants, woody plants, and herbaceous plants. Based on the order of Cd tolerance of the specific substrates [16,17,18,19] CC/CLXXXXCPC, XXXXXXCPC, CC/CLXXXXXXX, and XXXXXXXXX, we conducted a statistical analysis to identify research papers on the occurrence of PCR in species (Pubmed 1996–2025) [16,17,18,19] and analyzed of the PLAC8 domain on 27 June 2025 (https://www.ncbi.nlm.nih.gov/Structure/bwrpsb/bwrpsb.cgi, accessed on 27 June 2025). During evolution, gene duplication events have increased the number of *PCR* genes, which further facilitates family expansion in phylogeny. The difference in the numbers of CL/CC-XXX and XX-XXX indicates that *PCR* genes are forming new subpopulations and continuing to evolve, simultaneously generating differentially functional orphan genes with evolving functions (Figure 4).

## 4. Evolutionary Analysis of *PCR* Gene Family in Plants

**Figure 5:**
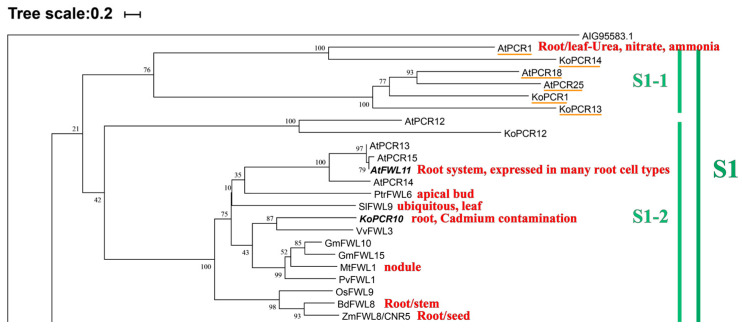
Local phylogenetic tree and cluster analysis of PCR family proteins. Through BlastP analysis, PCR family members were identified from the genome sequences of 12 different plant species. All identified protein sequences were queried in the Phytozome database (https://phytozome.jgi.doe.gov/, accessed on 12 June 2025; e-value ≤ 10^−19^, score ≥ 100) [68,91]. The Clustal W function in MEGA11 was used for comparison, and a Neighbor-Joining (NJ) method was employed in MEGA11 software to construct a phylogenetic tree, with 1000 bootstrap replicates [99]. A protein from the Acrocephalus schoenobaenus (AIG95583.1) was used as the root for the tree. The tree was divided into six clades, with the complete diagram presented in Figure S1. These clades are S1 (S1-1 incomplete group PCR, S1-2 focusing on 11 species FWL), S2 (leguminous and non-leguminous dicots), S3 (S3-1 monocots, S3-2 dicots and leguminous clustering), S4 (PCR expansion group, moss and angiosperm divergence group), S5 (MCA protein expansion and PCR conservation group), and S6 (moss and Arabidopsis divergence). We highlighted in red font the organs or tissues where each *PCR* gene is most abundantly expressed, as well as Cd tolerance.

The PLAC8 motif exhibits two distinct functions in plants. One is its role in controlling fruit size/weight and its inferred impact on fruit quality, which is directly related to plant yield and biomass [66]. For example, FW2.2 was first identified as a quantitative trait locus (QTL) and contributes up to 30% to crop yield, playing a key role in regulating crop yield and possessing a unique PLAC8 motif [67]. Based on these important findings, extensive research has been conducted on *PCR* gene homologs in leguminous plants and other economic crops, and studies have shown that these homologs exhibit functional conservation across different economic crops [68]. The other function of the PLAC8 motif is its function in conferring cadmium resistance and maintaining ion homeostasis in plants, playing a crucial role in the transport of heavy metals such as cadmium or zinc. These proteins are called the PCR family [19,20]. It should be noted that FWL and PCR share a high degree of sequence similarity and may belong to the same large CNR family [69]. Therefore, FWL may also be involved in heavy metal transport and accumulation, as supported by the relevant literature, with some scholars referring to it as a homolog of PCR. However, while the *PCR* gene family exhibits a certain degree of functional conservation, not all PCR proteins strictly adhere to this conserved feature [19]. Further research has revealed that various factors may contribute to functional differences, including but not limited to N/C-terminal sequence variations leading to the restructuring of the interactome, full-length differences affecting subcellular localization and complex assembly, partial or complete loss of functional motifs, and differences in substrate recognition specificity [70,71,72,73]. The *PCR* gene encodes a small protein (22 kDa) rich in cysteine residues [74], characterized by the presence of an uncharacterized PLAC8 motif consisting of two conserved cysteine-rich domains separated by a variable region, which is predicted to be a transmembrane segment [75]. Related literature suggests that the homolog of PCR, referred to as FW2.2, shares the same PLAC8 motif, transmembrane region, and membrane protein characteristics as the *PCR* gene, yet exhibits completely different functions, with the specific differences not being detailed [69]. Therefore, to thoroughly investigate these differences, we employed the Neighbor-Joining (NJ) method, which is computationally efficient, widely applicable, and robust to evolutionary rate heterogeneity, to construct the phylogenetic tree [76]. Using the latest plant genome sequences and their enhanced annotations [77], we analyzed 12 different plant species, namely *Arabidopsis thaliana* [78], *Physcomitrium patens* [79], *Glycine max* [80], *Medicago truncatulaj* [81], *Phaseolus vulgaris* [82], *Vitis vinifera* [83,84], *Solanum lycopersicum* [85], *Populus trichocarpa* [86], *Kandelia obovata* [87], *Oryza sativa* [88], *Brachypodium* distachyon [89], and *Zea mays* [90]. These selected species are economically important crops, covering different divisions of the plant kingdom, including one moss, three monocotyledonous plants, and eight dicotyledonous plants [91]. Most of the dicotyledonous plants are economically important or food crops, including mangrove plants from our previous research and Populus tomentosa, a species that plays a significant role in plant restoration. A total of 160 family members were identified (BlastP, e-value ≤ 10 × 10^−20^ and score ≥ 100; Supplementary Table S3) (Figure 5). Notably, several PLAC8 members were characterized for their significant roles in plant and fruit development in several monocots and dicots (marked in red) [92]. It is worth mentioning that *FWL* genes that characterize fruit size/weight negatively regulate overall plant size. Compared to other quantitative trait loci (QTLs) affecting tomato fruit weight, this gene serves as a major negative regulator of tomato fruit size, accounting for up to 30% of tomato fruit weight [67]. For instance, genes such as Cell Number Regulator 1 (CNR1; ZmFWL6) in maize [17], SiFW2.2 [93], and OsFWL3 [94] have been reported. The role of other *PLAC8* genes as negative regulators has also been documented in the pod-bearing tomato (Physalis floridana) [95] and soybean [96]. Simultaneously, several members of the plant cadmium resistance [45,96], ion homeostasis, and heavy metal transport, as well as Ca ion channels in dicotyledonous angiosperms [17,97,98], are marked due to their significant roles. Interestingly, although the CCXXXXCPC motif of these PCR members is partially incomplete, they still exhibit high expression under Cd stress. Recent studies have shown that, in addition to the CCXXXXCPC motif, there are cadmium stress binding sites in the transmembrane region [96]. We speculate that other key motifs exist within the PLAC8 motif, specifically located in the transmembrane region or within two domain regions.

The phylogenetic tree of multiple species reveals that these species are primarily distributed across six clades. Group S1 contains the most species, with 11 PCR proteins, but AtPCR12, KoPCR12, and six other more distant PCRs, along with 15 FWLs, are separately clustered, indicating a common ancestor but functional divergence during evolution. Group S1-2 contains four expanded PCRs that are relatively conserved with FWLs. KoPCR10 exhibits high cadmium tolerance, while the remaining ten are focused on FWL proteins. This group includes two sets of divergent functions of the PLAC8 motif. To explore commonalities and differences, we conducted motif analysis (MEME, 10 motifs) on 23 FWL/PCR proteins [100] (Supplementary Figure S2). We found that Group S1-1 is incomplete, and Group S1-2 remains incomplete (DPSJGWHGNVERQQR). However, starting from Group S1-2, all subsequent groups contain the PLAC8-specific motif QXXRELK in plants, which is absent in Group S1-1, which may be one of the reasons for its incompleteness. Surprisingly, AtPCR1/KoPCR14, which is closest to the outer group proteins, possesses the key substrate CCXXXXCPC, while the four *PCR* genes that are farther away and more conserved from PCR/FWL do not have the key motif CCXXXXCPC, further corroborating our hypothesis. Relevant literature has reported that the two substrates of this motif (CC\CPC) confer cadmium tolerance to plants, and the conservation of the motif varies, leading to different cadmium tolerance levels. The specific cadmium resistance substrate sequence in Arabidopsis thaliana is CC/CLXXXXCPC > XXXXXXCPC > CC/CLXXXXXX > XXXXXXXX [16]. Through sequence alignment analysis, we found that the proximity and distance of branches change accordingly, but there is no expected clustering change between incomplete and conserved groups on the evolutionary tree. Therefore, we speculate that there are still other key motifs present, and they may be located in transmembrane regions, at intracellular and extracellular binding sites, or within two TM helices. It is known that S5-1 is an MCA protein with two types of protein motif characteristic. The remaining distant proteins may be pseudogenes; see the Supplementary Tables S1–S4 for details. Motif alignment analysis revealed that all conserved PLAC8s contain the CCXXCAL motif, but S1-1 lacks it, which is a divergence point and may be the reason for its incompleteness. The CCXXCAL motif precedes the conserved plant motif QXXRELK and exhibits extremely high conservation. We first compared the motif QXXRELK and found no significant variations. Subsequently, we compared the CCXXCAL motif of S1-2 and discovered a polarization in the evolutionary tree, with drastic contraction and expansion starting from SiFWL10 (Supplementary Figures S1–S3), which confirms our hypothesis. Therefore, we speculate that CCXXCAL is another key substrate following CCXXXXCPC, but it is located near the QXXRELK motif. Reports in the literature indicate that mutants of this motif do not exhibit corresponding changes in cadmium resistance, which may be related to the CCXXCAL motif. Additionally, it is located in the transmembrane (TM) region. This is a novel discovery that provides theoretical support for subsequent related experiments. We will conduct wet experiments for further verification.

The PCR protein is a small transmembrane protein, with one domain at each of the N- and C-termini, both of which are embedded in the TM region and carry 1–2 transmembrane binding sites [42]. Topological predictions consistent with crystallographic validation indicate that this protein can form a pentamer by chelating with a metal model; amino acid substitutions alter substrate recognition and hydrophilicity, thereby regulating metal ion transport, constituting the core mechanism of its cadmium resistance [17]. Specifically, AtPCR1 forms a homopentameric structure with five TM1 helices surrounding a central pore with a diameter of 2.5–3 Å, while TM2 is anchored within the membrane. Conserved Cys/Glu/Asp residues within the channel form a metal coordination ring, while the Leu→Cys substitution in the CLXXXXCPC motif fine-tunes the pore diameter, enhancing selectivity for Cd^2+^/Zn^2+^. The pentameric cooperative conformational change (similar to the iris-like mechanism of CorA) facilitates Cd^2+^ capture–release transport driven by membrane potential and proton gradient [101]. Phylogenetic tree analysis and motif analysis show that PCR and FWL proteins carrying the PLAC8 motif are functionally distributed in series, but the heavy metal resistance mechanisms vary between proteins: a single base mutation in the transmembrane region can alter the efflux strategy. However, it remains unclear whether the PLAC8 motif is sufficient to explain the differences between the two distinct functions, and further experimental verification is needed.

Song et al. confirmed through point mutations that the deletion or replacement of the QXXRELK motif does not affect plant cadmium resistance [16,42]. This negative result suggests that in the PLAC8 protein, metal coordination function is still dominated by the CCXXXXCPC motif, while QXXRELK may mediate protein–protein interactions or subcellular localization rather than directly binding cadmium. Recent studies have found that OsFWL4 contains the CCXXG motif in the second transmembrane region, which can serve as an additional cadmium-binding site, significantly enhancing resistance. Structural models indicate that CCXXG induces transmembrane helix rearrangement, explaining the phenomenon where only four amino acids can induce a phenotypic leap [96]. The PLAC8 family has undergone subfunctionalization during evolution: traditional markers focus solely on the integrity of CCXXXXCPC, while the discovery of CCXXG provides new targetable alleles. Overlaying CCXXXXCPC with CCXXG holds promise for breeding higher and broader-spectrum heavy metal-tolerant strains. Current validation is limited to Cd and Zn stress; future studies could systematically assess the specificity and cross-talk of each motif under Pb, As, and pathogen-drought combined treatments to elucidate how the PLAC8 family achieves functional balance between metal homeostasis, biotic stress, and growth development.

From an evolutionary perspective, the differentiation of cadmium resistance in the PCR/FWL family follows three parallel trajectories: “expansion–reinforcement,” “contraction–conservation,” and “pseudogenization–loss.” The mechanisms and evolutionary signals underlying these trajectories can be clearly distinguished. In the expansion branch, rice OsFWL4 is currently the only member confirmed in vivo to specifically inhibit Cd root-to-stem transport. In addition to the conserved CCXXXXCPC motif, the newly emerged CCXXG motif in the second transmembrane region is reported for the first time as an additional cadmium-binding site, significantly enhancing resistance. The MCA subclass KoPCR6 fuses the Skp1 interaction domain to the C-terminal PLAC8 domain, forming a dual module of channel-regulation, thereby expanding cadmium efflux functionality. In contrast, soybean GmFWL13 retains only a 93-aa truncated ORF, lacking transmembrane regions and functional motifs, representing a typical pseudogenization event, directly confirming that this family simultaneously underwent functional gain and loss during evolution (Supplementary Figure S2). The contracted branch is characterized by reduced PLAC8 copy numbers but highly conserved sequences: AtPCR1 reduces Cd absorption through cytoplasmic chelation; AtPCR2 is localized to the plasma membrane and responds to cadmium stress through active efflux; AtPCR3 is highly homologous to AtPCR1/2 but has no obvious function due to extremely low expression levels (TPM < 0.5); rice OsFWL3/5/6 maintains steady-state resistance by downregulating Cd absorption flux. Experimental data further indicate that the contracting branch maintains stable Cd resistance under low copy number conditions; the expanding branch exhibits a dichotomy of high resistance and low expression–weak contribution due to motif innovation or domain recruitment.

## 5. Functional Differentiation of *PCR* Genes in Different Plant Types

### 5.1. Basic Functional Research in Model Plants

Arabidopsis thaliana, as a model plant, facilitates the study of *PCR* gene function [102,103]. Research has shown that *AtPCR1* in Arabidopsis thaliana exhibits different expression patterns in various tissues. Promoter–reporter gene constructs indicate that *AtPCR1* is expressed in stems, leaves, and flowers but not in roots, whereas *AtPCR2* is strongly expressed in roots and weakly expressed in leaves, stems, and flowers [43]. This expression difference allows *AtPCR2* to be responsible for the radial transport of zinc to the xylem in roots while reducing zinc accumulation in the surrounding medium, reflecting its dual function in roots. *OsPCR1* in rice also exhibits unique functions. *OsPCR1* is mainly expressed in the underground parts of rice and located in the plasma membrane, controlling grain size and Mg^2+^ accumulation. Studies have shown that the zinc hypersensitivity mediated by japonica-type *OsPCR1* is higher than that of indica-type *OgPCR1*, despite the fact that yeast cells expressing *OsPCR1* accumulate less zinc than those expressing *OgPCR1* [32]. This indicates that the C-terminal histidine residues of *PCR1* are important for zinc sensitivity, as all *PCR1* mutants displaying this histidine exhibit enhanced zinc sensitivity in yeast. Proteins with the conserved motif CCXXXXCPC also cannot fully determine cadmium resistance, and *AtPCR10*, despite having a complete substrate, has a relatively low overall expression level in plants, with higher expression levels only in guard cells and radicles [43]. Since this protein is predicted to be localized to the chloroplast, and yeast lacks homologous organelles, it is misdirected to the mitochondria, thereby spatially isolating it from the Cd detoxification core pathway (vacuolar sequestration, plasma membrane efflux). Furthermore, AtPCR10 is only weakly expressed in guard cells and young roots in plants, with protein abundance far lower than that of highly expressed members. Additionally, the heterologous system of yeast cannot mimic its post-transcriptional regulation, further weakening its transport activity. Compared to AtPCR1/2, AtPCR10 lacks the N-terminal extension and cytoplasmic tail segments which are believed to stabilize the pentameric conformation and regulate gating by binding to chaperone proteins or membrane microdomains. Their absence significantly reduces transport efficiency [104]. Therefore, while motif integrity ensures potential substrate binding capacity, localization, expression levels, and structural integrity collectively determine its Cd tolerance strength in yeast [105,106]. Sequence analysis reveals that, compared to *AtPCR1*, its CCXXCAL motif is incomplete.

### 5.2. Cadmium Accumulation and Tolerance Mechanisms in Hyperaccumulator Plants

The *SaPCR2* gene from *S. alfredii* was cloned from high-accumulation ecotypes. *SaPCR2* is highly expressed in roots but weakly expressed in shoots and non-hyperaccumulation ecotypes. It is localized to the plasma membrane and can reduce cellular cadmium content and enhance cadmium tolerance in Δzrc1 yeast. Heterologous expression in plants can reduce cadmium levels in roots. This is the first study on cadmium efflux mediated by a transporter protein in the hyperaccumulator *S. alfredii* [107]. This protein enhances cadmium tolerance by accelerating the efflux rate of Cd^2+^ from roots [24]. Another study showed that *SaPCR2* is highly expressed in the root elongation zone, and high-concentration zinc exposure downregulates its expression level in roots. *SaPCR2* can lead to zinc influx, and overexpression of *SaPCR2* significantly increases zinc uptake in roots, mainly distributed in the vascular buds, cortex, and epidermis, but it alters root morphology, inhibits meristem and elongation zone development, and reduces zinc accumulation in meristems [108]. Solanum nigrum, another common hyperaccumulator, follows the precedent of *S. alfredii* [109,110]. Although the function of the *PCR* gene has not been reported, its role in cadmium accumulation and tolerance is also noteworthy [111]. As a typical cadmium hyperaccumulator, it possesses five members of the CC/CLXXXXCPC and nine members of the XXXXXXCPC key substrates (Figure 4), with 33 PCR proteins (Supplementary Table S4), demonstrating the potential for further exploration of the application of transmembrane proteins in hyperaccumulators [112]. The *PCR* gene in Solanum nigrum may achieve efficient cadmium accumulation and tolerance by enhancing the adsorption capacity of the cell wall for cadmium and promoting vacuolar compartmentalization of cadmium. It has been reported that NcZNT1 in Noccaea caerulescens is considered a major root zinc/cadmium uptake transporter, highly expressed in the root epidermis and vascular bundles of roots and stems, suggesting its role in long-distance metal transport. Furthermore, two novel promoter regions of NcZNT1 have been identified as being potentially involved in the high expression of NcZNT1 and its regulation by plant zinc status [113]. This finding aligns with the role of the AtPCR2 protein as a zinc uptake and transport protein [43]. The Sedum zinc uptake and transport protein, also enriched in plants, has been previously reported. Through Blast alignment, it is highly likely that SstnPCR proteins with similar motifs can be identified, which holds significant practical importance for delving deeper into the role of transmembrane proteins in long-distance metal transport.

### 5.3. Cadmium Transport in Woody Plants and Potential for Ecological Remediation

In woody plants, members of the PCR/*PLAC8* gene family have emerged as central determinants of cadmium (Cd) flux and tolerance, positioning poplar and willow as model systems for phytotechnologies aimed at soil reclamation [114,115,116]. In Populus euphratica, heterologous expression of PePCR3 and PePCR2 in Saccharomyces cerevisiae conferred contrasting phenotypes: PePCR3 enhanced Cd tolerance without altering intracellular Cd content, whereas PePCR2 decreased cellular Cd burden. Both proteins localize to the plasma membrane and are transcriptionally up-regulated upon Cd exposure [117,118]. Consistent with the yeast data, transgenic P. euphratica lines overexpressing PePCR3 exhibited accelerated growth and greater biomass under Cd stress, whereas PePCR2-overexpressing plants displayed a 1.5-fold increase in root Cd^2+^ efflux relative to wild type, accompanied by elevated Cd translocation to aerial organs. Transcript profiling of PePCR3-overexpressing poplar revealed coordinated up-regulation of eight additional transporter genes, indicating that PePCR3 acts in concert with a broader transport network to redistribute Cd from sensitive cytosolic pools to the xylem and, ultimately, to harvestable stem tissues. By contrast, PePCR2 appears to function primarily as a plasma-membrane Cd-efflux pump, minimizing cytosolic toxicity while simultaneously facilitating Cd translocation. Beyond *PCR* genes, the PLAC8 subfamily also contributes to Cd homeostasis. In Populus, PcPLAC8-10 is strongly induced in Cd-treated roots; its ectopic overexpression increases net Cd^2+^ influx by 192% and total root Cd accumulation by 57%, underscoring its role in primary uptake [119]. In Salix linearistipularis, SlPCR6 resides at the plasma membrane of root epidermal and cortical cells and operates as an outwardly directed Cd^2+^ exporter, thereby limiting symplastic Cd entry and enhancing whole-plant tolerance [23]. Collectively, these findings illustrate a modular transport system in which plasma-membrane exporters (PePCR2, SlPCR6), putative sequestration facilitators (PePCR3), and high-affinity uptake carriers (PcPLAC8-10) orchestrate Cd absorption, translocation, and detoxification in woody species. Such mechanistic insight provides a robust molecular framework for engineering poplar and willow germplasm tailored to the phytoextraction or phytostabilization of Cd-contaminated soils.

### 5.4. Functional Expansion and Evolutionary Significance

*PCR* (Plant cadmium resistance) genes constitute a rapidly expanding gene family that has undergone recurrent lineage-specific amplifications across the plant kingdom. In the two principal model species—Arabidopsis thaliana and Oryza sativa—PCR proteins are predominantly implicated in cadmium (Cd) efflux and vacuolar sequestration, thereby governing the uptake, translocation, and ultimate partitioning of heavy metals. In A. thaliana, AtPCR2 operates within the root radial transport pathway, simultaneously facilitating the xylem loading of zinc (Zn) and restricting Zn accumulation in the external rhizosphere. O. sativa OsPCR1, by contrast, modulates grain size and magnesium (Mg) content via its influence on Zn and Cd fluxes, thus coupling micronutrient homeostasis to developmental processes. Lineages adapted to metalliferous soils exhibit marked expansions and functional differentiation. The Cd-hyperaccumulator Solanum nigrum harbors 33 PCR paralogues that diversified ~120 million years ago (Mya), coincident with early eudicot radiation. Transcriptomic and biochemical evidence indicates that S. nigrum achieves extraordinary Cd tolerance through two complementary mechanisms: (i) enhanced cell-wall adsorption that limits symplastic Cd entry, and (ii) efficient vacuolar compartmentalization that sequesters excess Cd away from sensitive cytoplasmic targets. Similarly, within the genus Sedum—another well-characterized hyperaccumulator—PCR proteins have been verified to function as Zn transporters and Cd efflux pumps, underscoring convergent molecular solutions to heavy metal stress. In woody taxa exemplified by Populus spp., *PCR* genes contribute to Cd uptake, xylem loading, storage, and detoxification, rendering these species attractive candidates for phytoremediation of contaminated sites. Comparative sequence analyses across angiosperms reveal that, with the exception of the weakly expressed AtPCR10, all PCR orthologues retain an intact CCXXCAL motif at the cytosolic N-terminus. Although this motif is strictly required for metal-binding activity in vitro, its in planta necessity awaits empirical validation. Collectively, the recurrent expansion and neofunctionalization of *PCR* genes reflect an adaptive evolutionary trajectory by which plants have fine-tuned metal homeostasis to meet the challenges of diverse edaphic environments.

## 6. Conclusions

This article presents a comprehensive overview of the pivotal role played by (Plant cadmium resistance, *PCR*) genes in the response to cadmium stress. It provides an in-depth analysis of their molecular mechanisms, functional differentiation, and unique expressions across various plant types, with special attention given to the functional expansion within the PLAC8 family. The small transmembrane proteins encoded by *PCR* genes effectively counteract cadmium toxicity through mechanisms such as efflux, chelation, transport, and compartmentalization, thereby mitigating the risk of cadmium transmission through the food chain. This offers a crucial theoretical foundation for addressing cadmium pollution issues.

*PCR* genes demonstrate significant variations among different plant types. In the model plants Arabidopsis thaliana and Oryza sativa, *PCR* genes are primarily involved in cadmium efflux and compartmentalization. In the hyperaccumulator Sedum alfredii, *PCR* genes function as cadmium (Cd) efflux and zinc (Zn) transport proteins for resistance. Despite the lack of reported *PCR* gene function in the cadmium hyperaccumulator Solanum nigrum, it possesses multiple crucial substrates and may achieve efficient cadmium accumulation and tolerance by enhancing the adsorption capacity of its cell walls for cadmium, facilitating root efflux, and promoting vacuolar compartmentalization of cadmium, thereby adapting to its survival requirements in high-cadmium environments. In the woody plant Populus, multiple *PCR* genes play a pivotal role in the processes of cadmium absorption, translocation, accumulation, and detoxification, rendering it a promising candidate for ecological restoration. This functional differentiation reflects the adaptive evolution of plants in different ecological environments. Notably, through multi-species phylogenetic trees and cluster analysis, we found that the *PCR* gene family has undergone significant differentiation across species, especially in group S1, which contains 11 species of *PCR* genes and exhibits differences between the S1-1 and S1-2 branches. Sequence alignment analysis revealed that the QXXRELK motif (the first 1-2 motifs of CCXXCAL) is unique to PLAC8 plants and has a significant impact on *PCR* clustering and branching, compensating for evolutionary differences and presenting distinctly differentiated groups and conserved groups. Moreover, reported cadmium-resistant *PCR* genes all contain this conserved motif, while some genes, such as *AtPCR10*, that are weakly expressed in whole plant tissues, are incomplete. More importantly, this motif is located at the transmembrane position. This discovery provides a reference for subsequent research on cadmium-resistant or transmembrane genes. These expansions not only augment the number of genes but also give rise to new subpopulations with diverse functionalities, including genes expressed in specific tissues and genes exhibiting varying cadmium tolerance capabilities. Functional expansion offers a genetic foundation for the adaptive evolution of plants in diverse ecological environments, empowering them to more efficiently address environmental stressors, such as cadmium contamination. It is worth emphasizing that, although cross-species evidence from Arabidopsis AtPCR1/2, rice OsPCR1, and others has preliminarily demonstrated the feasibility of *PCR* genes in cadmium-resistant crop breeding, the transition from laboratory to field application still requires overcoming critical validation stages. First, CRISPR/Cas technology should be used to introduce the optimal alleles into major cultivated varieties, and their comprehensive effects on yield, quality, and environmental adaptability should be systematically evaluated through multi-year, multi-location field trials. Second, multi-omics approaches such as transcriptomics, proteomics, and ionomics should be integrated to elucidate the expression regulatory networks of *PCR* genes and their interaction mechanisms with previously reported cadmium transporters. Regarding spillover effects, there is currently insufficient data: cadmium-resistant varieties may benefit neighboring crops by reducing soil cadmium concentrations, but they may also pose unknown ecological risks due to gene drift or changes in rhizosphere microbial communities. Therefore, future studies must include genetic monitoring and soil–plant–microbe interaction experiments to quantify potential benefits and risks, providing scientific basis for safe release.

Despite significant advancements in the study of *PCR* genes in plant cadmium resistance mechanisms, showcasing their immense potential for application, further in-depth exploration is still required for future research. For example, as small transmembrane proteins possessing a CCXXXXCPC cadmium resistance motif, can they form pentamers to facilitate transmembrane transport of heavy metal ions? What are the specific roles of hydrophilic and hydrophobic proteins, and can these be validated through molecular docking models and experimental evidence? Do all *PCR* proteins possess the potential to form pentamers, or is *AtPCR1* unique in this regard? These questions remain to be answered. Currently, our understanding of the molecular mechanisms of *PCR* genes in plant cadmium resistance, particularly their roles in signal transduction and gene expression regulation, remains shallow. Future research should further investigate the specific mechanisms and roles played by different types of *PCR* genes, proteins, and cadmium chelates in these processes. Through multi-omics approaches such as genomics, transcriptomics, and proteomics, more key genes and signaling pathways related to cadmium tolerance may be discovered. The uncharacterized motif of PALC8 imparts two distinct functions to plants. The literature has reported that *FW2.2* harbors a conserved CLXXXXCPC motif, whereas *PCR* contains a CC/CLXXXXCPC motif. Our findings reveal that the variation in the CCXXCAL motif enhances the distinction within the incomplete group and reinforces the conservation within the conserved group, potentially due to its impact on the expression of other amino acids. Further experimental validation is needed to confirm the specific mechanism. This discovery offers significant insights for the analysis of PLAC8 motif-associated genes, including *PCR*. In conclusion, *PCR* genes play an indispensable and crucial role in plant cadmium resistance mechanisms, offering promising prospects for research and application. Through further in-depth research and technological innovations, it is anticipated that more effective solutions will be provided for ecological remediation and sustainable agricultural development in the context of heavy metal pollution, thereby making greater contributions to safeguarding food security and human health.

## Data Availability

Data will be available on: https://figshare.com/s/35b5976f2cfb3c07402f (accessed on 27 June 2025).

## Supplementary Materials

The following supporting information can be downloaded at: https://www.mdpi.com/article/10.3390/biology14091163/s1, Figure S1: Phylogenetic tree and motif analysis of 12 species of PLAC8-related genes; Figure S2: Sequence Analysis of PLAC8 Proteins from 12 Species; Figure S3: Evolutionary Tree Expansion Analysis of CCXXCAL motif; Table S1: Annotation and amino acid sequences of 160 PLAC8 proteins identified in 12 different plant species. Table S2: *AtPCR* gene promoter targets transcription factors; Table S3: Annotations and amino acid sequences of 12 different plant PLAC8 proteins; Table S4: PCR Protein Family Members of Solanum nigrum.

## Abbreviations

The following abbreviations are used in this manuscript:

PCR	Plant Cadmium Resistance
FWL	Fruit Weight 2.2-Like
PLAC8	Placenta Associated 8
CAX	Cation Exchanger
TM	Transmembrane
ROS	Reactive Oxygen Species
O_2_^−^	Superoxide Anion Radical
H_2_O_2_	Hydrogen Peroxide
·OH	Hydroxyl Radical
MRE	Metal Response Element
GSH	Glutathione
PCs	Phytochelatins
MT1C	Metallothionein-1C
CorA	Cobalt resistance A
CNR	Cell Number Regulator
MCA	Mid1-Complementing Activity
Cys	Cysteine
Glu	Glutamic acid
Asp	Aspartic acid
SKP	Sphase Kinase-Associated Protein
