# Supplementary material for: Research Progress on PCR (Plant Cadmium Resistance) Genes in Plants
Source: Biology (Basel). 2025 Sep 1;14(9):1163. doi: 10.3390/biology14091163 (PMC12467093; doi:10.3390/biology14091163)

Supplementary Figure S1 Phylogenetic Tree of PLAC8 Proteins in 12 Species

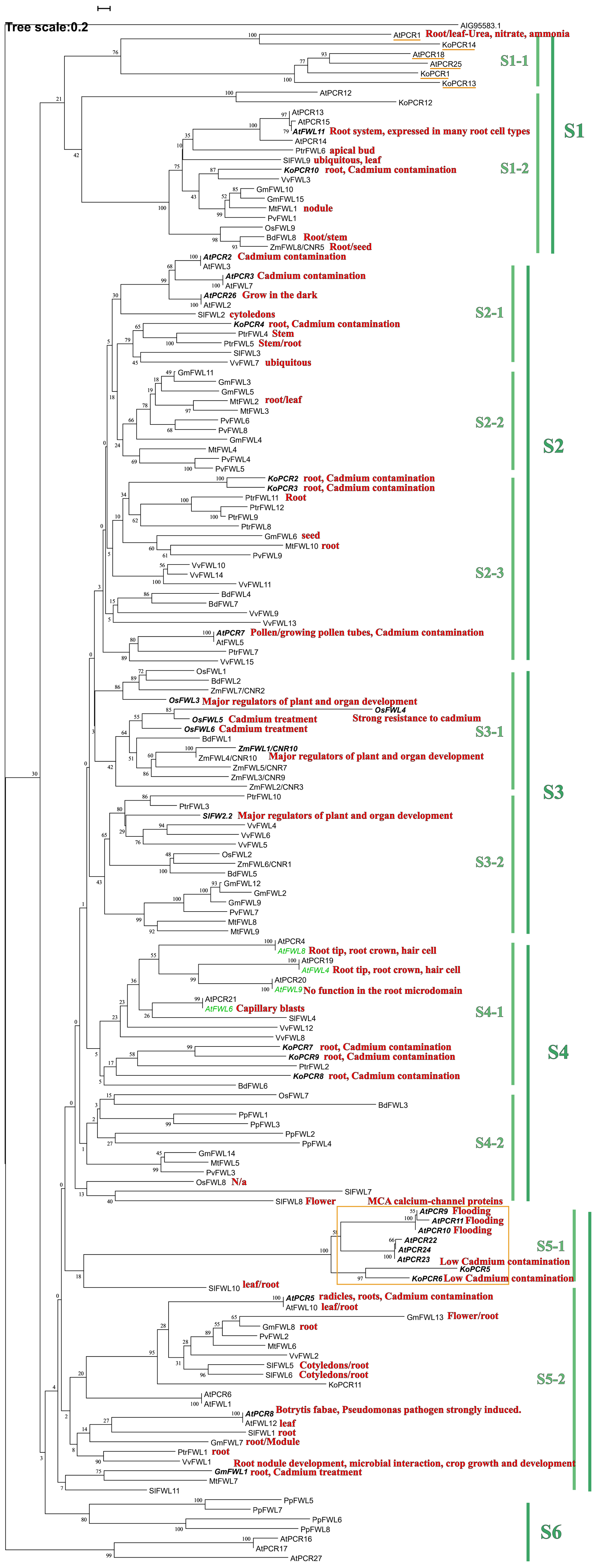

Supplementary Figure S2 Sequence Analysis of PLAC8 Proteins from 12 Species

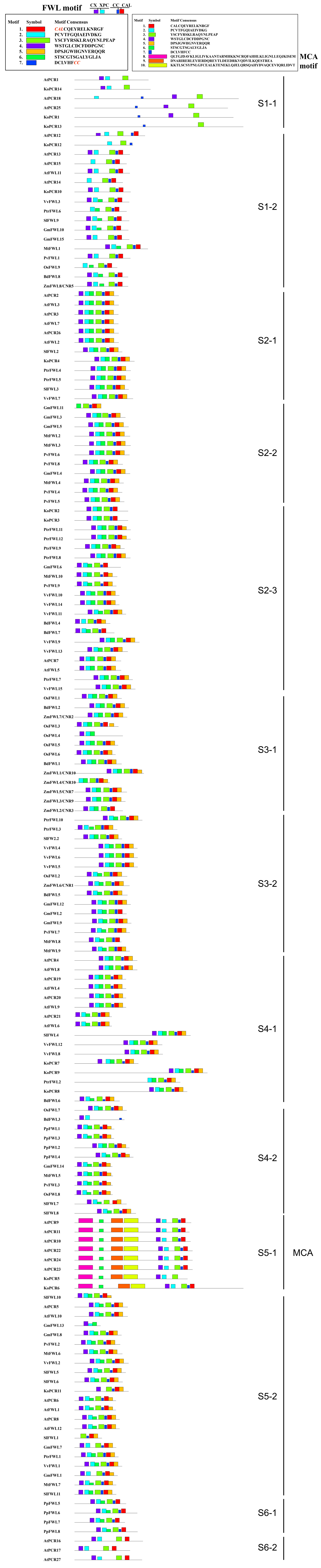

Supplementary Figure S3 Evolutionary Tree Expansion Analysis of CCXXCAL motif

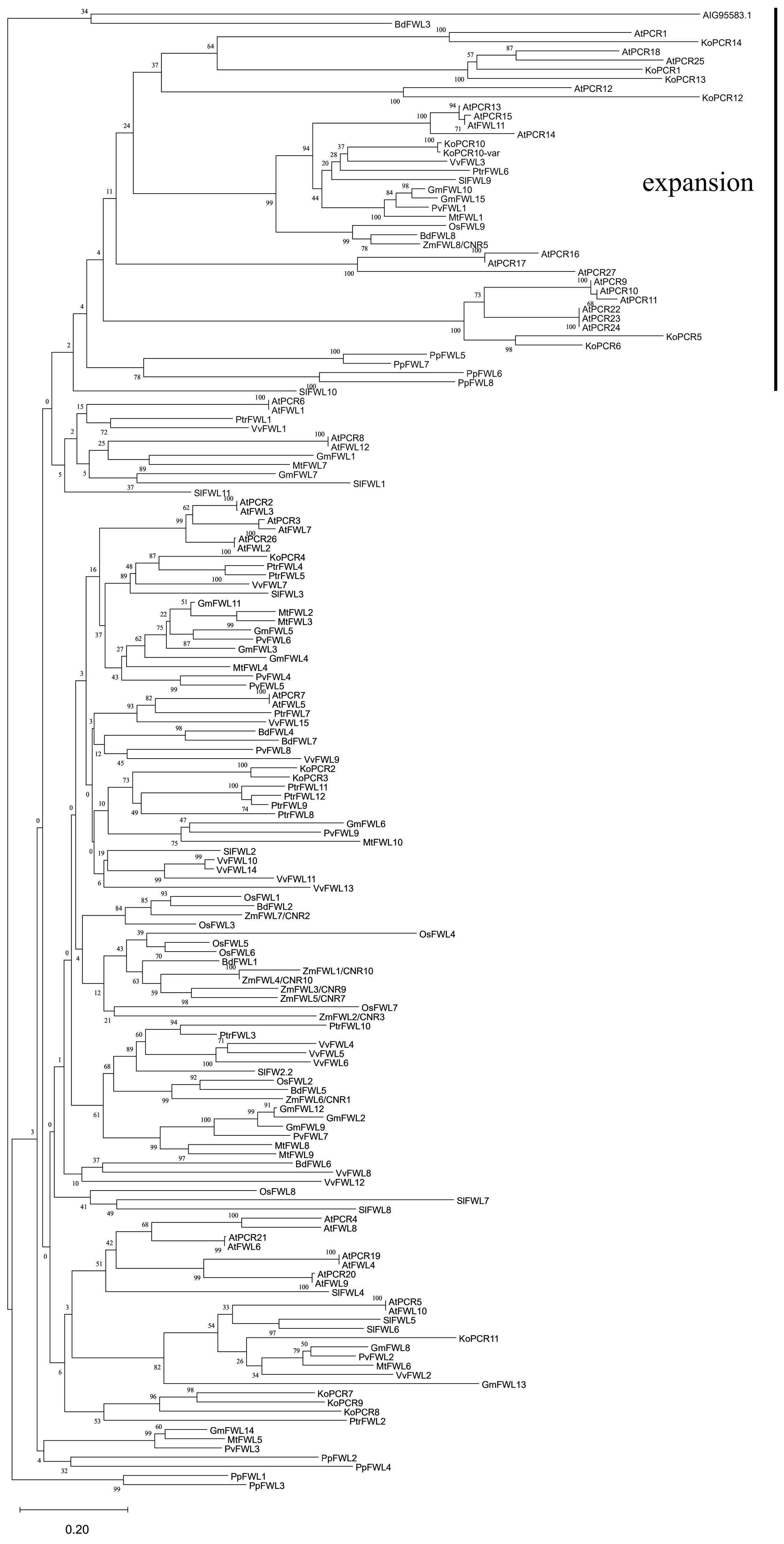

Supplement: Supplementary file 1 [file Supplementary figure.pdf › Supplementary figure.pdf]
